# Judgments of effort exerted by others are influenced by received rewards

**DOI:** 10.1038/s41598-020-58686-0

**Published:** 2020-02-05

**Authors:** Max Rollwage, Franziska Pannach, Caedyn Stinson, Ulf Toelch, Igor Kagan, Arezoo Pooresmaeili

**Affiliations:** 1 Perception and Cognition Group, European Neuroscience Institute Göttingen (a Joint Initiative of the University Medical Center Göttingen and the Max-Planck-Society), Göttingen, Germany; 2 Wellcome Trust Centre for Human Neuroimaging, University College London, London, United Kingdom; 3 Max Planck University College London Centre for Computational Psychiatry and Ageing Research, London, United Kingdom; 4 Biological Psychology and Cognitive Neuroscience, Freie Universität Berlin, Berlin, Germany; 5 Decision and Awareness Group, Cognitive Neuroscience Laboratory, German Primate Center – Leibniz Institute for Primate Research, Göttingen, Germany; 6 Leibniz ScienceCampus Primate Cognition, Göttingen, Germany

**Keywords:** Social behaviour, Cooperation

## Abstract

Estimating invested effort is a core dimension for evaluating own and others’ actions, and views on the relationship between effort and rewards are deeply ingrained in various societal attitudes. Internal representations of effort, however, are inherently noisy, e.g. due to the variability of sensorimotor and visceral responses to physical exertion. The uncertainty in effort judgments is further aggravated when there is no direct access to the internal representations of exertion – such as when estimating the effort of another person. Bayesian cue integration suggests that this uncertainty can be resolved by incorporating additional cues that are predictive of effort, e.g. received rewards. We hypothesized that judgments about the effort spent on a task will be influenced by the magnitude of received rewards. Additionally, we surmised that such influence might further depend on individual beliefs regarding the relationship between hard work and prosperity, as exemplified by a conservative work ethic. To test these predictions, participants performed an effortful task interleaved with a partner and were informed about the obtained reward before rating either their own or the partner’s effort. We show that higher rewards led to higher estimations of exerted effort in self-judgments, and this effect was even more pronounced for other-judgments. In both types of judgment, computational modelling revealed that reward information and sensorimotor markers of exertion were combined in a Bayes-optimal manner in order to reduce uncertainty. Remarkably, the extent to which rewards influenced effort judgments was associated with conservative world-views, indicating links between this phenomenon and general beliefs about the relationship between effort and earnings in society.

## Introduction

Every action comes at a cost and we constantly evaluate whether an effortful behaviour was “worth it”. Effort judgments are particularly important in a social context as effort is one of the core dimensions for evaluating behaviour of others. For instance, people  who contributed more effort to a cooperative task are perceived as more likeable^1^, while free-riders, who do not contribute to the joint endeavour, are punished^2,3^. Effort estimations also influence group dynamics and broader social attitudes, for instance, the support for welfare benefits is strongly linked to the perception of whether the people who receive those benefits have made an effort to find a job^4^. Although retrospective effort estimations have an important impact on different aspects of our lives, decision neuroscience has so far mainly focused on prospective effects of expected effort on action plans. The common finding of these studies is that effort is processed as a value discounting factor that is traded off against potential rewards when making choices for oneself ^5–9^ as well as for others^10^. However, decision-making often goes beyond an immediate comparison between costs and benefits and involves evaluating a stream of actions and outcomes that occur over time^11^. As such, effort judgments at any given moment inherently involve a retrospective evaluation of the exerted effort in the past and the resultant individual or social outcomes. Surprisingly, the underlying mechanisms of retrospective evaluations of effort have been underexplored in cognitive neurosciences. In comparison, social psychology has extensively investigated retrospective evaluations of effort and the interactions between attribution of rewards and effort depending on the social context (e.g. attribution theory^12^ and effort justification^13^). For instance, it has been shown that people differ in the attribution of success when evaluating their own behaviour (where success is attributed to more effort^14^) or others’ behaviour (where success is attributed to luck^12,15^). However, to understand the underlying mechanisms of such interactions during decision-making, a computational approach is required where the contributions of rewards and effort to the outcome evaluations are analytically modelled^16–18^.

A recent study by Pooresmaeili and colleagues began to fill this gap by using a computational approach to investigate different contributing factors that influence retrospective effort evaluations^19^. Intuitively, people might have the impression that they hold accurate representations about the effort they, and others, have spent. In contrast to this intuition, in this study self-judgments of effort were influenced by the magnitude of obtained rewards: the same level of effort was rated differently depending on the reward, reflecting a bias in effort estimations. However, it is not known whether the influence of reward on effort estimations is limited to self-judgments (i.e. representing a form of self-serving bias^20,21^) or whether reward information is invariably incorporated when judging exerted effort in other contexts as well.

Interestingly, in the study of Pooresmaeili *et al*.^19^ the integration of rewards into effort judgments depended on the contingency between reward and task difficulty: reward had a larger influence when it had a strong association with the task difficulty, and hence, provided reliable probabilistic information about the exerted effort. Thus, the integration of reward into effort ratings resembled Bayesian cue integration^22–24^, according to which different sources of information are combined, weighted by their reliability, to infer the “true” state of the world. In such a paradigm, the consideration of rewards provides an additional source of information for estimating the exerted effort, rather than an irrational bias. This might be especially true when judging another person’s effort, because of a stronger inherent uncertainty. In this case, people do not have access to the internal, interoceptive feelings of exertion and therefore can only make judgments based on less reliable externally observable information – e.g. how much time was spent on a task. Therefore, we predict an enhanced influence of reward on judgments about others’ effort compared to self-judgments.

The weighting of different information sources by their reliability might be further influenced by prior beliefs about the relevance of each information source. For instance, there is a wide range of individual differences regarding assumptions about the relationship between effort and success/reward. A potential driver of those individual differences could be a Protestant work ethic which is often associated with conservative world-views^25^. Moreover, the belief that success/reward is mainly driven by investment of effort might lead to justifying economic hierarchies and contribute to stabilization of existing inequalities^19,26,27^. Such beliefs and their ensuing attitudes show a considerable overlap with political conservatism as it has been proposed that preference for social stability and acceptance of social inequalities are two core aspects of conservative world-views^28,29^. We therefore hypothesized that the extent to which reward influences effort estimations in a simple task might be related to broader conservative attitudes.

**Figure 1:**
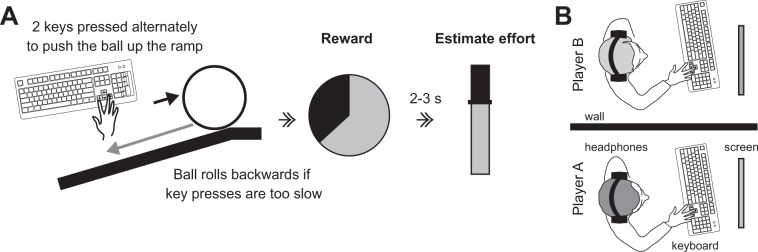
Experimental setup and task. (**A**) Participants were asked to move a ball up a ramp by performing fast, alternating key presses. A gravity force was simulated, displacing the ball backwards by a constant amount on each display frame. We used six levels of task difficulty, corresponding to the amount of ball displacement per frame. After the ball was successfully pushed all the way to the top of the ramp, participants received a monetary reward, where the reward amount was contingent upon task difficulty (the actual reward value was drawn from Gaussian distributions with means ranging from 1.5 to 6.5 cents and a standard deviation of 1.2 cents). Subjects rated their effort by shifting the position of a vertical sliding bar. In half of the trials participants performed the task themselves and rated their own effort. In the other half, participants rated the other person’s effort after watching them performing the task. **(B)** Subjects were seated next to each other in the same room, separated by a partition wall. When the partner was pressing the keys to push the ball, subjects saw on their screen how the ball was moved up the ramp and heard via the headphones a beep tone for every key press the other person was conducting.

In the current study, we asked pairs of subjects to perform an effortful sensorimotor task, interleaved with a partner, and to rate either their own or the partner’s exerted effort (see Fig. 1). The task varied in difficulty (6 difficulty levels) and after each trial, but before the effort rating, both participants were informed about the obtained reward. We investigated whether participants similarly integrated reward magnitude into their effort judgments for self- and other-judgments, and whether this incorporation of reward information would be in line with the principles of Bayesian cue integration for both judgment types. Secondly, we tested the hypothesis that the incorporation of rewards into effort judgments might be associated with conservative attitudes.

## Methods

### Participants

The final sample included 51 subjects (28 females, age 20–38 years, mean 26.97, SD 3.95 years). The primary aim of our study was to investigate a group level effect for the influence of reward on effort ratings. However, we also wished to investigate individual differences with regard to the relationship between reward integration and conservative attitudes. We therefore had to balance group homogeneity (advantageous for investigating group level effects) versus heterogeneity (advantageous for investigating individual difference correlations). Thus we decided to acquire a homogenous sample in terms of education level – consisting of 92% university students – while we also ensured that a certain amount of diversity existed amongst our participants since they were from ~25 different fields of studies and included both German and English native speakers (86% German), with 64% working part-time (versus unemployed or exclusively studying). Our sample also proved to contain a reasonable degree of diversity with regards to the conservative world-views (self-report of political orientation – ranging from 0 = very liberal, 100 = very conservative – mean 42.3, SD 21.9).

Eleven subjects were excluded from the original sample (N = 62) as there was strong indication of random effort ratings (but we note that when not excluding any participants the results are qualitatively similar, see Supplementary Information for results of the full sample). Specifically, task difficulty was the experimental factor controlling required effort, thus subjects should show an association between task difficulty and their effort ratings. Therefore, 11 subjects whose effort ratings were not significantly influenced by task difficulty (i.e. no significant effect of a trial-by-trial linear regression between task difficulty and effort ratings, see Supplementary Fig. S1) were excluded. Participants were paid 8 Euro per hour, plus a bonus based on their performance in the experiment, resulting in a total mean payment of 20.14 ± 1.8 Euro. All subjects gave written informed consent before taking part in the experiment after all the procedures were clearly explained to them. The study was conducted in full accordance with the Declaration of Helsinki and was approved by the local ethics committee of University Medicine Goettingen, proposal number **15/7/15**.

The sample size was determined by an *a priori* power analysis which was based on the effect sizes of Pooresmaeili and colleagues^19^. The targeted sample size (N = 62) gave us power of 99% to replicate the influence of reward on self-judgments, 77% power to detect a small to medium difference between self- and other-judgments (Cohen’s d = 0.35) and 77% power to detect at least a medium correlation between our behavioural effects and conservative world-views.

### Experimental design

The behavioural experiment was adapted from a previous study^19^ in order to test two subjects simultaneously, and lasted around 2 hours. Participants pressed two keys of the keyboard, as fast as possible, to push a ball up a virtual ramp before rating the exerted effort (see Fig. 1A). Trials were presented interleaved between both subjects. In half of the trials participants performed the task themselves and rated their own effort (self-judgment). In the other half, participants rated the other person’s effort (other-judgment) after watching them performing the task. Each participant in a pair performed 200 trials resulting in a total of 400 trials which were grouped into 10 experimental blocks (each containing 40 trials).

While the participants tried to push the ball up the ramp with fast alternating key presses, the ball rolled back by a constant amount during each frame of the display, simulating a gravity force. This gravity force differed pseudo-randomly between trials and created the six difficulty levels of the task (individually adjusted for each participant, see below). If the participants managed to push the ball up the ramp within 10 seconds they were rewarded for this successful trial (only the participant who performed the trial was rewarded). The amount of reward was contingent on the task difficulty and the actual reward value was drawn from Gaussian distributions with means ranging from 1.5 to 6.5 cents and standard deviation of 1.2 cents. In most of the trials (70%) the reward was presented before participants rated the effort, but in some of the trials (30%) the reward was presented *after* participants had judged the effort. Trials in which reward was shown after the estimation of effort served as a reference, since in those trials no influence of reward was present.

The subjects were seated inside the same room, next to each other, separated by a partition wall (Fig. 1B). Participants met face-to-face and were introduced to each other before the experiment. Hence, they had convincing information that they were actually rating the effort of another person during the experiment. On the screen, participants observed the ball moving up the ramp, independent of whether they performed the task themselves or whether the other person was playing. Moreover, subjects wore headphones and each key press (theirs or their partner’s) was accompanied by a beep tone, such that they had visual and auditory information about the key presses on every trial. In trials where the participants conducted the task themselves they judged their own exerted effort and in trials when the other person performed the task, they estimated the effort that the other person had performed. Subjects did not see their partner’s ratings, in order to prevent reciprocal influences.

### Stimuli and task

The stimulus presentation and registration of behavioural responses was controlled by one personal computer. Stimuli were produced with MATLAB and the Psychophysics Toolbox^30^. Each trial consisted of a stimulus (ball and ramp), a reward, and an effort rating display all shown on a black background (a trial timeline is shown in Fig. 1A). The stimulus display contained the ball (radius: three visual degrees), initially positioned at the leftmost (starting) part of the ramp (ramp length: 19 visual degrees; both ball and ramp had a light grey colour). The ball was displaced up the ramp with consecutive alternate key presses (left and right arrow keys) until it reached the upper plateau. Each key press resulted in a constant amount of displacement (0.87 visual degrees per key press) and was counteracted by a gravity force of variable strength that displaced the ball backward. Moreover, each key press caused a beep tone which was played on the headphones of both participants. The gravity force levels for each subject were determined individually at the beginning of the experiment by having each subject perform two trials in which they pushed the ball up the ramp by pressing both keys alternately and consecutively as fast as possible. These trials were coded in a way that faster key presses adaptively increased the difficulty of the task. Thus the amount of key presses was not limited to a specific upper plateau – for every participant the task was adaptively adjusted to match their maximum possible speed of key presses. To motivate the participants during these trials, they were told that they would receive a 1 Euro bonus if they managed to push the ball up the ramp. In the main experiment, 90% of the gravity force necessary to counteract the maximum number of key presses in a limited time (10 s) averaged across these two trials determined the maximum gravity force. Five further equally spaced gravity levels were calculated at subsequent 10% steps (80%, 70%, 60%, 50%, 40%). This estimation was done for each participant alone; the other participant of the pair waited outside the room.

During the main experiment, trials were aborted if key presses did not occur fast enough (maximum pause allowed was 2 s). If participants were able to successfully push the ball to the top of the ramp, they received a monetary reward, with the amount contingent on task difficulty. Reward magnitude was defined based on six Gaussians with means of 1.5, 2.5, 3.5, 4.5, 5.5, and 6.5 cents with a SD of 1.2 cents. Based on the difficulty level of the trial the reward was drawn from one of these Gaussian distributions (e.g. for difficulty level six the reward was drawn from the Gaussian with mean of 6.5 cents). Thus, over all trials reward was related to difficulty levels (as the difficulty determined the mean from which the reward was drawn), but within one difficulty level the reward was randomly drawn from a Gaussian distribution. Participants were not explicitly informed about this relationship but had to infer this contingency through learning (see Supplementary Information for additional information). The reward display consisted of a pie chart that depicted subjects’ reward as a proportion of maximum reward possible and a number that showed the reward in Arabic numerals. The effort rating display consisted of a slider, and participants were instructed to set the slider at a position that represented their perceived effort during a trial. Specifically, participants were informed that they should regard the maximum of the effort scale as the maximum effort level they could experience in the task and that they should rate their effort proportionate to this maximum level. In 70% of trials, the reward display was shown immediately after the stimulus display, whereas in 30% of trials it occurred after the rating of effort. A subset of trials (20% of all trials, randomly chosen) included an additional colour-report task at the end of the trial. In this task, subjects were asked to report whether they had seen a brief (21 ms, three display frames) colour change on the ball (to green, red, or blue), which occurred in 50% of the trials. The colour-report task served two purposes. Because task difficulty and reward were the only parameters that varied across trials, we intended to distract participants from the main purpose of this experiment to prevent ad hoc strategies of relating reward and effort. Moreover, this task ensured that subjects followed the movements of the ball on the screen (both when performing the task themselves as well as when observing the other participant). This attention check showed that participants indeed engaged in the task and paid equal attention to the screen for self- and other-trials (performance accuracy: mean ± SD: 68% ± 14% and 69% ± 12% for self and other respectively; accuracy in both cases was significantly higher than 50% chance level as verified by Wilcoxon signed rank test, p < 0.001).

Before the actual experiment started, participants conducted 36 training trials (which were not analysed) to become familiar with the experiment and to learn about the effort-reward contingency. We did not explicitly inform participants about the effort-reward contingency in order to avoid demand characteristics that could have prompted participants to pay exaggerated attention to the relationship between rewards and the task (see the Supplementary Information for additional analysis indicating that these contingencies were indeed learned).

### Questionnaires

Prior to the experiment (24 hours before the behavioural experiment), participants completed online questionnaires about social attitudes and political orientation. We used a political orientation self-report (on a scale from 0 = “very liberal” to 100 = “very conservative”) to assess explicit conservative orientation. As indirect indicators for conservatism we also assessed social dominance orientation^31^, reflecting a general preference for hierarchical intergroup relations, and right-wing authoritarianism^32^, measuring authoritarian submission, authoritarian aggression, and conventionalism^33^. Since we were interested in the underlying factors linking these scales, we conducted a factor analysis over the scales, using maximum-likelihood estimation. The number of factors was based on the Scree-test^34^ (see Supplementary Fig. S2), which indicated that one factor was sufficient to explain the interrelation between these questionnaires. Factor scores of the first factor were extracted and used to correlate political orientation with the behavioural measures.

Since conservatism, especially right-wing authoritarianism and social dominance orientation, have been reported to correlate negatively with empathy^31,35^, and empathy could arguably have an impact on our behavioural effects of interest, we assessed empathy and perspective-taking as covariates^36^.

### Statistical analysis

All presented results were tested with two-tailed tests. Results from regression analysis are always reported as standardized betas. Outlier values of effort ratings (3 standard deviations above or below the participant’s mean effort) were removed from the analysis, since some subjects reported in the debriefing to have occasionally rated extreme effort values to test their assumptions about the aim of the experiment (e.g. whether they could influence the received reward by their effort ratings). However, when not excluding any effort ratings from the analysis the results were qualitatively similar (see Supplementary Information for results without any excluded effort ratings).

For the analysis of the relationship between trial-to-trial variation in reward magnitude and effort ratings, both were z-scored within difficulty levels, resulting in values representing variation in reward magnitude and effort rating with respect to the average of the corresponding difficulty levels. After having created these within difficulty variations we pooled them across all difficulty levels and conducted a robust linear regression (robust regression toolbox in MATLAB) with the reward as predictor and effort ratings as the dependent variable.

For comparison of the computational models, each model was fitted to participants’ effort ratings, separately for self- and other-judgments, and Bayesian Information Criterion (BIC) scores^37,38^ were calculated. For model comparison the sum of individual BIC scores was used.

To investigate the relationship between conservative world-views and the influence of rewards on effort estimations, linear regression analysis was used. Scores of the first factor from the factor analysis were used as an indicator for conservative world-views. The z-scored sum of slopes for self- and other-judgments between reward magnitude and effort estimations was used as a model-free indicator for the influence of rewards on effort estimations. As a model-based measure of reward integration, we used the sum of reward weights predicted by the Bayesian average model (ω_*r-self*_ + ω_*r-other*_). These were positively correlated (r = 0.36, p = 0.01), indicating a general tendency to integrate rewards into effort-ratings independent of whether judging oneself or another person. The influence of rewards on effort estimations was used as the predictor and conservative-world views as the dependent variable in the linear regression analysis, controlling for empathy and perspective-taking as covariates.

## Results

### Reward magnitude influences effort estimations for self- and other-judgments

We investigated whether people integrate reward magnitude when judging the effort another person has exerted. Participants performed an effortful task interleaved with a partner and rated either their own or the other partners effort (Fig. 1). We first investigated the influence of reward magnitude on effort estimations by regression analysis of trial-to-trial variation in reward magnitude and effort estimation. This analysis yields a model-free measure of the influence of reward on effort ratings (model-free slope). The analysis was conducted separately for the self- and the other-judgments of each participant and only trials in which reward information was presented before the effort rating were considered here (70% of trials). The regression slopes (β_model-free_) were calculated between the trial-to-trial variation of reward (reward_within-difficulty variation_: rewards were z-scored within each difficulty level) and estimated effort (Effort_within-difficulty variation_: effort ratings were z-scored within each difficulty level).

1 $${{\rm{E}}{\rm{f}}{\rm{f}}{\rm{o}}{\rm{r}}{\rm{t}}}_{{\rm{w}}{\rm{i}}{\rm{t}}{\rm{h}}{\rm{i}}{\rm{n}}{\textstyle \text{-}}{\rm{d}}{\rm{i}}{\rm{f}}{\rm{f}}{\rm{i}}{\rm{c}}{\rm{u}}{\rm{l}}{\rm{t}}{\rm{y}}{\textstyle \text{-}}{\rm{v}}{\rm{a}}{\rm{r}}{\rm{i}}{\rm{a}}{\rm{t}}{\rm{i}}{\rm{o}}{\rm{n}}}={\beta }_{{\rm{m}}{\rm{o}}{\rm{d}}{\rm{e}}{\rm{l}}{\textstyle \text{-}}{\rm{f}}{\rm{r}}{\rm{e}}{\rm{e}}}\ast {{\rm{r}}{\rm{e}}{\rm{w}}{\rm{a}}{\rm{r}}{\rm{d}}}_{{\rm{w}}{\rm{i}}{\rm{t}}{\rm{h}}{\rm{i}}{\rm{n}}{\textstyle \text{-}}{\rm{d}}{\rm{i}}{\rm{f}}{\rm{f}}{\rm{i}}{\rm{c}}{\rm{u}}{\rm{l}}{\rm{t}}{\rm{y}}{\textstyle \text{-}}{\rm{v}}{\rm{a}}{\rm{r}}{\rm{i}}{\rm{a}}{\rm{t}}{\rm{i}}{\rm{o}}{\rm{n}}}+\varepsilon $$

**Figure 2:**
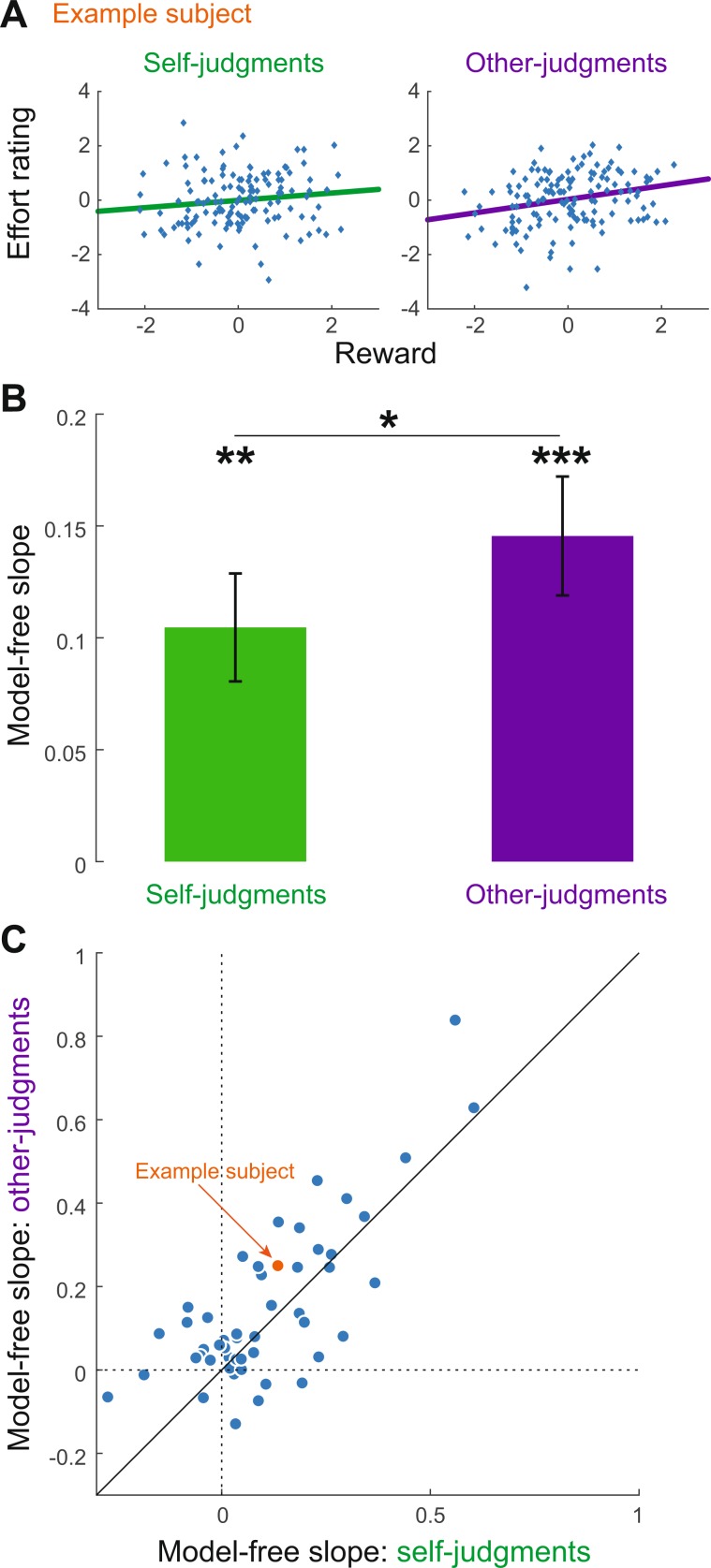
Influence of reward on effort judgments for self and others. In order to estimate the influence of reward on effort judgments, the trial-by-trial fluctuations in rewards were used in a linear regression to predict estimated effort, separately for self- and other-judgments of each subject. (**A**) Data of one representative subject is shown as a scatter plot and a linear regression between trial by trial variation in reward and effort for self- (left) and other-judgments (right). Trial-by-trial reward magnitudes and effort ratings were z-scored within each difficulty level, representing variations in comparison to the average of the difficulty level. (**B**) Group average of the model-free slope ± standard error of mean are presented (N = 51). (**C**) The individual model-free slopes for self- (x-axis) and other-ratings (y-axis) are presented as a scatterplot. The diagonal line represents the line of equality; a point on this diagonal would indicate that a subject shows the same amount of reward integration for self- and other-judgments. The example subject in (**A**) is indicated by an orange dot. One-sample signed test and Wilcoxon signed rank test, two-sided *p < 0.05, **p < 0.01, ***p < 0.001.

Higher rewards were associated with higher effort ratings (Fig. 2A,B), everything else being constant. Across subjects, there was a significant influence of reward variation on effort estimations for self-judgments (one-sample signed test, p < 0.001, Cohen’s d = 0.57, Fig. 2B) as well as for other-judgments (one-sample signed test, p < 0.001, Cohen’s d = 0.73, Fig. 2B). Interestingly, the effect of rewards on effort estimations was significantly stronger for other- than self-judgments (mean slopes of 0.15 and 0.1 respectively, Wilcoxon signed rank test, p = 0.015, Fig. 2C). Nevertheless, the amount of reward integration (i.e. the model-free slope measure) for self- and other-judgments was highly correlated across subjects (r = 0.76, p < 0.001; Fig. 2C), indicating that integrating reward information into effort judgments is a general tendency of individuals, independent of the type of required judgment. We obtained similar results when we controlled for slight differences between actual gravity forces of trials that belonged to the same difficulty level (see Supplementary Information).

### Computational modelling reveals that reward information and perceived level of exertion are combined in a Bayes optimal manner

**Figure 3:**
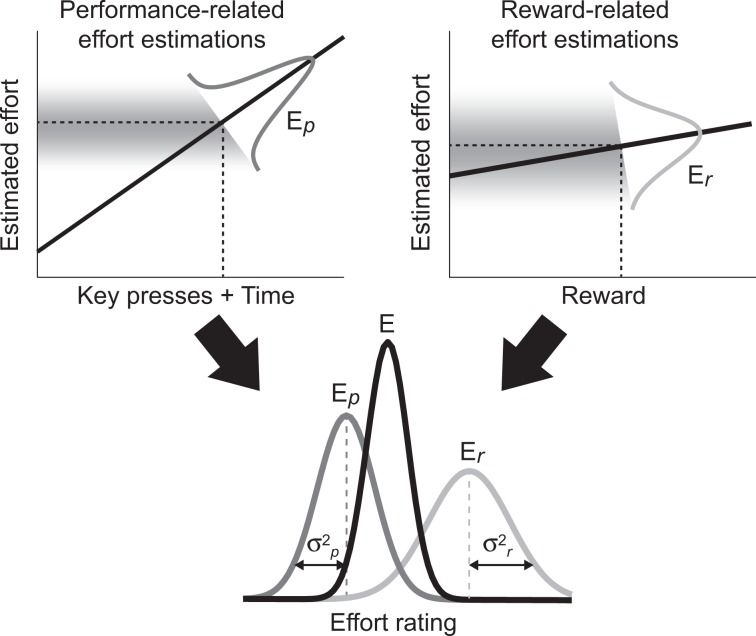
Multiple information sources are integrated to form a final effort rating. Two types of information are combined to derive the final effort estimation (E): E_*p*_, the effort estimation based on performance-related sensory information (measured by amount of key presses and trial duration), and E_*r*_, the effort estimation based on reward-related information. Both estimates are also subject to noise, and will differ by some degree. Bayes optimality predicts that effort ratings will be best described by the Bayesian average model, where the influence of each signal on E is weighted by its reliability (ω_*p*_ and ω_*r*_), represented by the width of the distributions (σ^2^_*p*_ and σ^2^_*r*_).

Having established an influence of reward magnitude on self- and other-judgments of effort, we next investigated how rewards were integrated with other information sources related to the exerted effort to form the final effort ratings. This computational approach allowed us to explore the mechanisms underpinning the influence of reward on effort ratings (*cf*. Fig. 2). To this end, we compared the predictions of five computational models with the behavioural data. These models focussed on the manner in which two informative cues related to the level of exertion, i.e. performance-related information (E_*p*_) and reward information (E_*r*_), are combined to form the final effort rating (Fig. 3).

**Figure 4:**
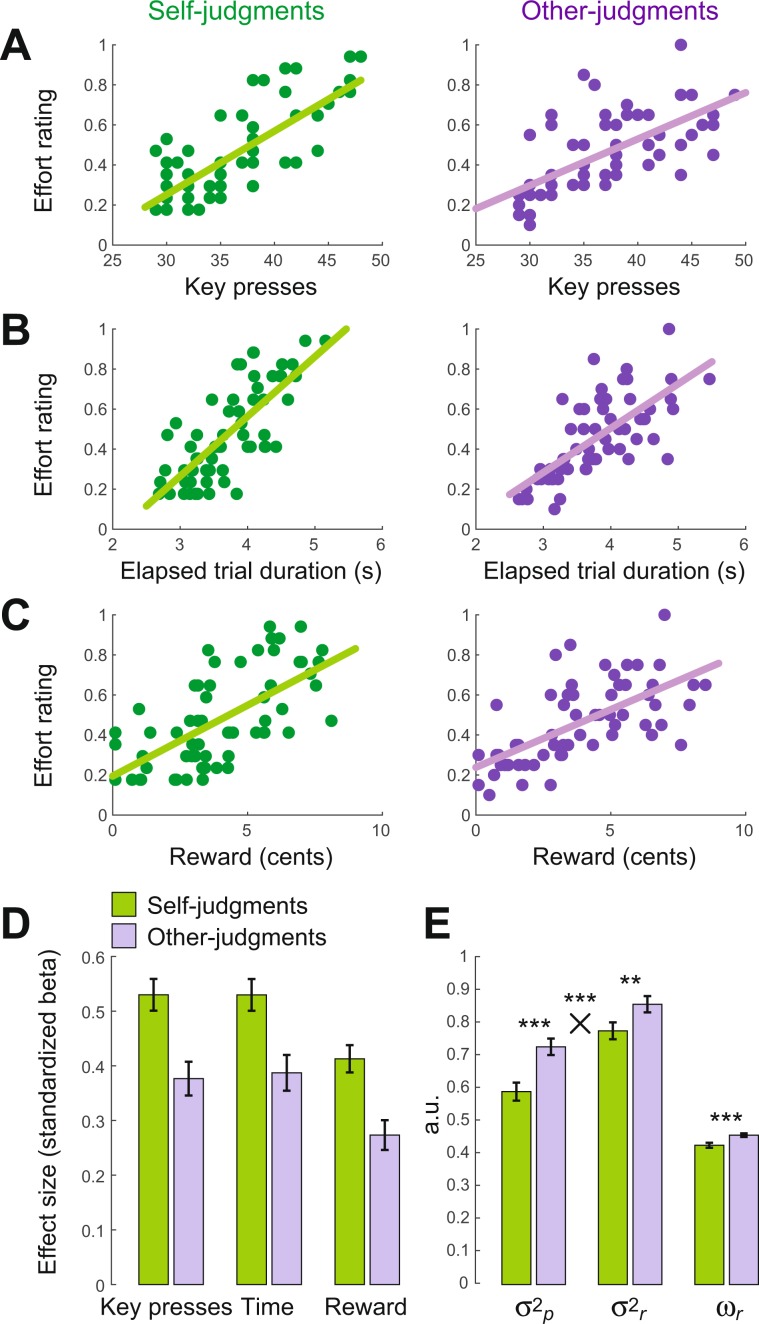
Performance-related and reward-related effort estimates for self-and other-judgments, used in computational models. (**A–C**) Scatter plots showing the data of one representative subject. See Supplementary Fig. S4 for the data of all participants. (**A**) Relation between the number of key presses and effort ratings for self- (left) and other-judgments (right), based on the trials in which effort was rated *before* the reward was revealed (30% of all trials). The regression lines show β_*k*_ for this participant. (**B**) Similar to (**A**) for the relation between trial duration and effort ratings. (**C**) Similar to (**A**) for the relation between reward magnitude and effort ratings. Note that this relation effectively captures the correlation between reward and effort ratings built-in by the task: more difficult trials were associated with higher rewards and were subjectively rated with higher efforts, therefore reward was related to the subjective estimations of exerted effort in these trials. (**D**) Group average ± standard error of the mean of the relationship between effort ratings and key presses (β_*k*_), elapsed trial duration (β_*t*_) and reward (β_*r*_), for self- and other-judgments. Note that the relationships between all task variables (key presses, time and reward) and effort ratings are higher for self-judgments, which is expected as in general people have more reliable estimates of their own effort. (**E**) Group averages ± standard error of mean of ${{\rm{\sigma }}}_{p}^{2\,}$, ${{\rm{\sigma }}}_{r}^{2\,}$ (derived from Eqs. 2–5) and ω_*r*_ (derived from Model 4). In line with panel D, ${{\rm{\sigma }}}_{p}^{2\,}$ and ${{\rm{\sigma }}}_{r}^{2\,}$ values were higher for other- compared to self-judgments. This indicates that all the task variables were less reliable predictors of effort judgments for others compared to self-judgments. However, this effect was more pronounced for the performance-related criteria (key presses and time) than for rewards (two-way repeated measures ANOVA with factors self/other and performance/reward): the main effect of self vs. other F(1,50) = 16.743, p = 0.0002; the interaction F(1,50) = 16.743, p = 0.0002). Therefore, reward was a relatively more reliable predictor of effort-ratings for other- than self-judgments. Thus, the model-based reward weighting parameter (ω_*r*_) predicted by the Bayesian average model was higher for other- than self-judgments, in line with model-free results shown in Fig. 2.

The first informative cue, referred to as E_*p*,_ provides an estimation of the expended effort based on observable, performance-related information as measured by the amount of key presses and the duration of a given trial. These quantities were observable by subjects in every trial, both for self- and other-judgments, albeit potentially with greater saliency on self-judgment trials. As perceived exertion is expected to increase with increased physical output and trial duration, E_*p*_ models the participants’ subjective effort estimation using objective performance-related information (as shown in Fig. 4A,B,D, see also Supplementary Fig. S3). We can leverage this to deduce participants’ presumed internal effort representations based on such observable quantities. Thus, we can define the expected effort estimation given performance-related information (key presses and elapsed trial time) as follows:

2 $${{\rm{E}}}_{p}={{\rm{\beta }}}_{{0}p}+{{\rm{\beta }}}_{k}\ast {\rm{key}}\,{\rm{presses}}+{\beta }_{t}\ast {\rm{time}}+{{\rm{\varepsilon }}}_{p}$$

where β_*0p*_, β_*k*_ and β_*t*_ were fitted to the trials in which the effort was rated *before* the reward was revealed (30% of all trials), giving an estimation of effort judgments that is unaffected by reward information.

The second informative cue referred to as E_*r*_ reflects the degree to which exerted effort could be inferred from rewards. Based on the task design, rewards were contingent on task difficulty, and thus on exerted effort (Fig. 4C,D). In other words, rewards carried probabilistic information regarding difficulty levels (i.e. the actual reward for each trial was drawn from a Gaussian distribution around a certain mean). Due to this relationship, there was a correlation between rewards and effort, as more difficult trials yielded higher rewards and also led to higher effort judgments. Importantly, this correlation was present in all trials, including those where effort was rated *before* reward presentation. Therefore, we can use these trials to quantify the degree to which effort judgments (E_*r*_) can be predicted based on reward as follows:

3 $${{\rm{E}}}_{r}={{\rm{\beta }}}_{{0}r}+{{\rm{\beta }}}_{r}\ast {\rm{reward}}+{{\rm{\varepsilon }}}_{r}$$

Analogously to Eq. (2), we derived β_*0r*_ and β_*r*_ from the trials in which the effort was rated *before* the reward was revealed. Although in these trials subjects were not exposed to the rewards at the time of the effort rating, their effort judgments were still affected by the task difficulty. Therefore, we can use β_*0r*_ and β_*r*_ to capture the theoretical information that rewards convey regarding the perceived difficulty (i.e. mean difference of reward between difficulty levels) and hence exerted effort, but not the actual impact of reward on effort judgments. The error term (ε_*r*_), represents the (un)certainty (e.g. due to some overlap of rewards between the different difficulty levels) with which exerted effort can be theoretically inferred from rewards.

Both effort estimations, E_*p*_ and E_*r*_, are associated with some uncertainty, representing how well exerted effort can be inferred from those different sources. The uncertainty associated with E_*p*_ and E_*r*_ can be formalized as the mean squared error:

4 $${{\rm{\sigma }}}_{p}^{2\,}=\frac{1}{n}\mathop{\sum }\limits_{i=1}^{n}{{\rm{\varepsilon }}}_{p}^{2}$$

and

5 $${{\rm{\sigma }}}_{r}^{2\,}=\frac{1}{n}\mathop{\sum }\limits_{i=1}^{n}{{\rm{\varepsilon }}}_{r}^{2}$$

where n is the number of 30% of trials where effort was rated before the reward was revealed.

We then fixed β_*0p*_, β_*k*_, β_*t*_, β_*0r*_, β_*r*_, ${{\rm{\sigma }}}_{p}^{2\,}$ and ${{\rm{\sigma }}}_{r}^{2\,},$ and applied them to the trials in which effort was rated *after* the reward was revealed (70% of trials), to get a trial-by-trial estimation of subjects’ internal effort representation based on either performance related information (E_*p*_) or reward information (E_*r*_) only. In each of these trials, E_*r*_ and E_*p*_ can differ by some amount (ΔE ≠ 0). We consider several models of how these two informational sources might be integrated into the actual effort rating E.

**Model 1** (performance only model) assumes the actual effort rating is only based on the E_*p*_ without taking reward information into account. Thus, the E_*p*_ would directly result in the actual effort rating E:

6 $${\rm{E}}={{\rm{E}}}_{p}$$

**Model 2** (reward only model), in contrast, only relies on reward information (E_*r*_) and completely ignores performance information:

7 $${\rm{E}}={{\rm{E}}}_{r}$$

**Model 3** (simple average model) assumes that both sources of information are considered and that E is an equally weighted average of E_*p*_ and E_*r*_:

8 $${\rm{E}}=0.5\ast {{\rm{E}}}_{p}+0.5\ast {{\rm{E}}}_{r}$$

So far, the models did not take the reliability of the information into account. However, **Model 4** (Bayesian average model) assumes that the variance of the different sources of information is used in order to weight E_*p*_ and E_*r*_ in a Bayes optimal way (see Fig. 3):

9 $${\rm{E}}={\omega }_{p}\ast {{\rm{E}}}_{p}+{{\rm{\omega }}}_{r}\ast {{\rm{E}}}_{r}$$

where ω_*r*_ = 1/σ^2^_*r*_/(1/σ^2^_*r*_ + 1/σ^2^_*p*_) and ω_*p*_ = 1 − ω_*r*_. The variances σ^2^_*r*_ and σ^2^_*p*_ are derived as explained above (Eqs. (4) and (5)) and the weights are directly derived from variances. Therefore, models 1–4 contain no free parameters.

Moreover, it might be possible that E_*r*_ and E_*p*_ are combined, but with a different weighting than predicted by any of the previous models. Thus, in **Model 5** (flexible weighting model) we set the weighting parameter ω_*r*_ as a free parameter and ω_*p*_ = 1 − ω_*r*_. In contrast to Model 4, in Model 5 ω_*r*_ and ω_*p*_ are not directly derived from the estimated variances.

The model evaluation was done by computing the maximum-likelihood fits to the trial-to-trial data of individual subjects, separately for self- and other-judgments. The quality of fits was compared by the Bayesian Information Criterion (BIC)^37,38^.

The model comparison revealed that for both, self- and other-judgments, the Bayesian average model had the lowest BIC (Table 1), showing that this model provides the most accurate and parsimonious account of the data. For self-judgments the second best model was the flexible weighting model, whereas for other-judgments it was the simple average model.

**Table 1:** Results of model comparison. BIC: Bayesian Information Criterion. BIC values are summed across 51 subjects. The Bayesian average model (bold font) had the lowest BIC score and is therefore ranked highest by the model comparison. ∆BIC: Difference in BIC of each model minus the BIC of the best fitting model (BIC_model_ - BIC_best_).

Model	Self-judgment			Other-judgment		
	BIC	∆BIC	Median R^2^	BIC	∆BIC	Median R^2^
1. Performance only	14908.6	+70.2	0.316	16078.3	+310.7	0.193
2. Reward only	16246.2	+1407.8	0.194	16402.1	+634.4	0.092
3. Average	14952.9	+114.4	0.306	15777.4	+9.7	0.196
4. Bayesian average	**14838.4**	**0**	**0.336**	**15767.7**	**0**	**0.205**
5. Flexible weighting	14855.6	+17.2	0.362	15868.3	+100.6	0.218

These results provide additional evidence that reward information is integrated when forming effort estimations about self and others. Moreover, subjects integrate different sources of information weighted by their reliability, as predicted by Bayesian cue integration. Therefore, the integration of rewards into final effort ratings appears optimal in terms of information usage as rewards are combined with performance-related information based on the reliability with which each information source can be used to infer the exerted effort.

Importantly, the Bayesian model can account for the differential integration of rewards for self- and other-judgments (*cf*. Fig. 2B). Indeed, the model predicts that rewards should be assigned with a higher weight (i.e. ω_*r*_ as in Eq. (9): model-based reward weighting) during other- compared to self-judgments (mean ± SD: ω_*r-self*_ 0.43 ± 0.05; ω_*r-other*_ 0.46 ± 0.04; Wilcoxon signed rank test, p < 0.001, see Fig. 4E). Crucially, this model not only predicts a difference between self- and other-judgments, but also provides a mechanistic explanation for why this difference should occur. In line with theoretical assumptions that participants should have more uncertainty when judging other people’s effort, we found that ${{\rm{\sigma }}}_{p}^{2\,}$ was indeed higher in the other- compared to self-judgments (Wilcoxon signed rank test, p < 0.001, see Fig. 4E). This suggests that the stronger incorporation of rewards into other-judgments is indeed due to the inherently higher uncertainty for estimates of another person’s exertion level.

### The extent of reward incorporation into effort judgments is linked to conservative world-views

**Figure 5:**
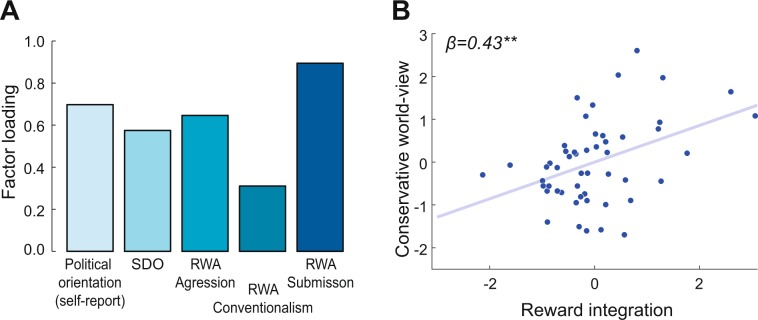
Conservative world-views (factor scores) are positively correlated with the amount of reward integration into effort estimation. (**A**) A factor analysis over multiple questionnaires revealed that one factor (based on Scree-test), which can be interpreted as conservatism, explained the interrelation between these scales. SDO: social dominance orientation; RWA: right-wing authoritarianism. (**B**) Stronger influence of rewards on effort estimations is positively associated with conservative world-views. The z-scored sum of the model-free slopes for self- and other-judgments (slope_self_ + slope_other_) is used as a model-free indicator of reward integration. Since we controlled for empathy and perspective taking in the regression analysis, we present values of reward integration from which influences of these covariates are regressed out (N = 51). Linear regression, two-sided **p < 0.01.

After having demonstrated that reward influences the estimation of exerted effort of self and others, we asked whether this behavioural effect showed any meaningful relationship with broader social attitudes. We hypothesized that over-reliance on reward information in effort estimations could be associated with acceptance of economic inequalities and reluctance to social change, as these attitudes might represent a prior assumption about strong links between effort and reward/wealth. In order to probe a relationship between our behavioural effects and broader social attitudes, we assessed several questionnaires related to conservative world-views^31,32^ (see Methods) and conducted a factor analysis on these scales using maximum-likelihood estimation. Based on the Scree-test^34^ we identified a sharp drop in eigenvalue after the first component (see Supplementary Fig. S2), indicating that one factor, explaining 76% of the variance, was the best and most parsimonious solution for the covariance structure of the questionnaire data. We interpreted this factor as “*conservative world-views*” based on the factor loadings (Fig. 5A). This factor represented acceptance/support of economic inequality, reluctance to social change and obedience to established authorities.

We extracted factor scores for every subject and correlated them with our behavioural effect of reward integration into effort judgments (with both the model-free slope as well as the model-based reward weighting parameter). Due to the high correlation between the model-free slope for self- and other-judgments (cf. Fig. 2C), we used the sum of both effects (from the model-free analysis: slope_self_ + slope_other_) as an indicator for the extent to which subjects were biased in their effort estimations by rewards.

In line with our hypothesis, subjects with stronger influence of reward magnitude on effort estimations displayed more conservative world-views (β = 0.43, p = 0.003, see Fig. 5B), underlining an interesting link between this behavioural effect and social attitudes. Importantly, this association between conservatism and reward influence could also be shown when separately considering self-judgments (β = 0.33, p = 0.02) and other-judgments (β = 0.46, p = 0.001).

We then asked whether the reward integration in conservative participants followed the principles of a Bayes optimal model where the weight assigned to the reward is proportional to the uncertainty in performance-related effort estimates (i.e. σ^2^_*p*_ in Eq. (4)). In order to test this hypothesis we performed two follow-up analyses. Firstly, we calculated the correlation between each individual’s model-based reward weighting parameter (ω_*r*_ = ω_*r-self*_ + ω_*r-other*_) derived from the Bayesian average model and the score on conservative attitudes. Indeed, more conservative participants showed higher ω_*r*_ parameters (β = 0.39, p = 0.005), suggesting that they integrated rewards more strongly due to higher uncertainty regarding the effort a person has exerted (the same result held true when looking separately at ω_*r-self*_: β = 0.33, p = 0.015 and ω_*r-other*_: β = 0.31, p = 0.03). However, if the degree of reward integration in this group was fully accounted for by a Bayes optimal strategy, the direct effect of the model-free slope measure of reward integration (slope_self_ + slope_other_) on conservative attitudes should disappear when controlling for the effect of ω_*r*_, as the model-based weighting parameter (ω_*r*_) would fully capture the reward influences that are Bayes optimal (i.e. adjusted based on the degree of uncertainty in performance-related effort estimates). Intriguingly, this was not the case. When simultaneously entering the model-free slope measure and the model-based reward weighting parameter as predictors of conservative attitudes, we found separate significant influences of ω_*r*_ (β = 0.42, p < 0.001) and the model-free slope measures of reward integration (β = 0.46, p < 0.001). This indicates that the strength of the influence of reward information on effort judgments in conservative participants is over and above the predictions of the Bayes average model.

It is remarkable that an association between a low-level behavioural task and high-level social attitudes could be found. In particular, the size of this effect is surprising (R^2^ = 0.33, using the model-free and model-based measures of reward integration as simultaneous predictors) since the influence of reward explained the variance of conservative attitudes at least to a similar extent as well-established socio-demographic predictors such as age, gender, education or income^39–41^.

## Discussion

We used retrospective effort evaluations during a sensorimotor task to show that judgments about our own and other people’s exerted effort are influenced by received reward magnitude, with more pronounced effects for other-judgments. Computational modelling revealed that both judgment types were constructed in a Bayes optimal manner, weighting performance-related information and rewards by their reliability (inverse of variance). This model explains the increased integration of rewards for other-judgments as an optimal strategy to compensate for the inherently higher uncertainty in judging someone else’s effort. Furthermore, the extent to which individuals were influenced by rewards when judging efforts was positively correlated with conservative world-views. The relationship between conservatism and the strength of reward integration was partially, but not completely, explained by a Bayes optimal strategy, pointing to other contributing factors.

Both self- and other-effort judgments were influenced by received rewards, showing that this effect is not an instance of self-serving bias but instead represents a general cognitive process of information usage. Importantly, the influence of rewards was even stronger when judging someone else’s effort. This finding is contrary to predictions from psychological attribution theory that success (i.e. high reward) is more strongly attributed to internal factors (i.e. exerted physical effort) for self-judgments compared to other-judgments^42^. In contrast, other studies have found that people incorporate outcomes less when making judgments about themselves as compared to others^43^, which is in line with our findings. In those studies the differential incorporation of outcomes for judging own and others’ behaviour has been described as an *introspection illusion*^43–45^ suggesting that people put too much weight on subjective internal information when evaluating themselves, deviating from optimality. Our results however do not support the interpretation that people necessarily weigh internal and external information sub-optimally during self-assessment. Using computational modelling, we showed that people weigh internal (subjective representations of exertion level based on key presses and trial duration) and external (reward) information in a statistically optimal fashion where the weight of each information source is determined by its reliability. This complements recent findings showing that updating of decisions in a social context follows Bayes optimal principles^46^. Furthermore, it follows from Bayes optimal integration of information that subjects should incorporate rewards more when judging other people’s effort because they have less reliable estimates of others’ performance-related criteria. Thus, our results build on and extend a previous body of work on how people attribute outcomes for self or others to external and internal causes, providing a computational framework that helps to understand the cognitive processes underlying asymmetries in attribution of outcomes for self vs. others.

In our study, rewards were contingent upon performance and hence provided a reliable source of information regarding the expended effort. Therefore, it was rational to use rewards in order to infer the level of effort and our modelling results show that this was accomplished through Bayes optimal integration of information. While Bayesian integration of information has been shown to underlie many aspects of decision-making, our study reveals its link to judgments of effort made in a social context for the first time. The Bayesian framework predicts that the integration of rewards into effort estimations should be limited to situations when reward is indeed a valid cue for exerted effort (i.e. reward is contingent on task difficulty and thus correlated with effort). This assumption is supported by findings of a previous study^19^ where we included a condition in which rewards were random/unrelated to task difficulty. In this condition participants did not show an influence of rewards on effort estimations for self-judgments.

At the same time, it is known that the human brain is extremely sensitive to reward acquisition^47^, and decision-making agents are irrationally biased to repeat choices that recently led to reward outcomes even when such outcomes were dissociated from long-term informational value^48^. Therefore, it would be important to investigate if the receipt of a monetary outcome such as used in our study, has stronger influence than would be explained by the purely informational content of the reward feedback (or any other abstract task-contingent feedback).

The weighing of different relevant information sources by their reliability is the optimal way of combining information. In such a scheme, increased incorporation of reward information occurs when effort estimates based on performance criteria are less reliable. We propose that inferring the precision of internal representations of effort based on the actual physical exertion level (here modelled as the amount of key presses and elapsed time) is a form of metacognitive ability^49^ enabling humans to accurately monitor and report their mental states. It would be interesting to probe whether an improvement of this metacognitive ability (e.g. by training) might result in a reduced incorporation of reward information when rating one’s own effort, as would be predicted by the Bayesian framework.

The finding that more conservative attitudes correlated with greater influence of rewards on effort estimations shows that the behaviour in a basic sensorimotor task might generalize to more global attitudes with potential societal ramifications. This resembles our recent study showing that people holding radical political beliefs showed reduced metacognitive abilities, as measured in a low-level perceptual task^50^. While we are convinced that laboratory tasks in conjunction with computational modelling provide critical insights for gaining a mechanistic understanding of the cognitive and neural processes underpinning (political) attitudes^51,52^, we also acknowledge the limitations of our approach. Due to the time consuming nature of our psychophysical task, the sample in our study was relatively small and of reduced diversity (predominately German students). However, the strength of the relationship between conservative views and our behavioural effects indicates the robustness of this effect. On the other hand, exposure to a Protestant work ethic could be a factor influencing our behavioural results, making our findings particularly relevant for western societies where the debate on meritocracy is particularly intense^53^.

Our data indicate that conservative people integrate reward more into effort ratings, partly due to higher uncertainty in estimating effort based on other information sources. Interestingly, there is a solid body of literature suggesting uncertainty aversion as one of the core psychological foundations of conservative attitudes (see^29^ for an extensive review). It might be promising to revisit those existing psychological findings by using a computational approach to see whether findings about uncertainty aversion could be accommodated within a Bayesian framework, to facilitate examining the neurocognitive underpinnings of social and political attitudes^54,55^. Importantly however, the increased effect of reward in more conservative participants was not fully explained by the increased uncertainty of effort estimates. In fact, conservative participants integrated rewards over and above the extent predicted by a Bayes optimal model. We suggest that this might reflect the contribution of other socio-economic factors such as a stronger prior belief regarding the relationship of reward and effort in our society, and a pursuit of deterministic rationalization.

What are the societal ramifications of the finding that more conservative people exhibit a stronger influence of rewards when judging their own and especially other people’s effort? We surmise that in some situations, such exaggerated influence of reward can lead to a biased perception of people’s exerted effort. Our results suggest that people with conservative views might judge other people’s effort more through the received gains, indicating that in everyday life they might adhere to the view that people with low income have also exerted less effort, and conversely, prosperous people deserve their wealth since they have worked harder, perhaps ignoring other contributing factors. Our fine-grained analysis enables a better understanding of the cognitive building blocks contributing to such societal attitudes.

In summary, we have shown that retrospective judgments about the amount of exerted effort are affected by obtained rewards, both for oneself as well as for the others. While reward magnitudes are often explicit in naturalistic environments, effort demands are rarely explicit and are usually learned by trial and error in the course of successive actions^56^. An important direction for future research is to determine how retrospective evaluations regarding the internal states and the state of the environment could in turn influence prospective value- and effort-based decisions, thus relating the effects we observed to their potential adaptive role in shaping human choices in individual and social contexts.

## Supplementary information

Supplementary Information.

## Data Availability

The datasets generated and analysed in the current study are available from the corresponding authors upon request, and will be uploaded to Open Science Framework data repository (https://osf.io/).
