# Supplementary material for: Judgments of effort exerted by others are influenced by received rewards
Source: Sci Rep. 2020 Feb 5;10:1868. doi: 10.1038/s41598-020-58686-0 (PMC7002752; doi:10.1038/s41598-020-58686-0)
Supplement: Supplementary file 1 — Supplementary Information. [file 41598_2020_58686_MOESM1_ESM.pdf]

Judgments of effort exerted by others are influenced by received rewards – Supplementary Information

Max Rollwage[1,2,3,*], Franziska Pannach[1], Caedyn Stinson[4], Ulf Toelch[4], Igor Kagan[5,6,¤], Arezoo Pooresmaeili[1,6,*,¤]

[1] *Perception and Cognition Group, European Neuroscience Institute Göttingen (a Joint Initiative of the University Medical Center Göttingen and the Max-Planck-Society), Germany*

[2] *Wellcome Trust Centre for Human Neuroimaging, University College London, London, United Kingdom*

[3] *Max Planck University College London Centre for Computational Psychiatry and Ageing Research, London, United Kingdom*

[4] *Biological Psychology and Cognitive Neuroscience, Freie Universität Berlin, Berlin, Germany*

[5] *Decision and Awareness Group, Cognitive Neuroscience Laboratory, German Primate Center (DPZ), Göttingen, Germany*

[6] *Leibniz ScienceCampus Primate Cognition, Göttingen, Germany*

[*] Correspondences should be addressed to:

Max Rollwage max.rollwage.16@ucl.ac.uk

or

Arezoo Pooresmaeili a.pooresmaeili@eni-g.de

[¤] IK and AP are joint last authors

Learning the contingency between difficulty levels and rewards

Participants performed 36 training trials (which were not analysed) to familiarise themselves with the experiment and to learn about the difficulty-reward contingency. The number of training trials was based on our previous work[19]. We had no explicit checks whether the reward-difficulty contingency was learned. The rational for this was that any additional check could trigger subjects to pay excessive attention to these contingencies, and thereby confound our results. Furthermore, Bayesian cue integration does not require an explicit representation of the correlation between cues[22,23] and explicit reasoning may even hamper Bayesian inference and cue integration[24]. Importantly however, if participants had not learned this contingency, at least implicitly, rewards would have not influenced effort ratings. Thus, the presence of our observed effect is an indicator that participants learned these associations (even if sub-consciously/implicitly).

Moreover, to double check that the reported effects were stable during the course of the experiment, we examined whether there was any change over time in the degree to which reward was integrated into effort ratings. For this purpose, we split our data into the first and the second half of the experiment. If subjects were still learning the contingency of reward and difficulty during the main experiment, the influence of reward on effort ratings would have been weaker in the first half of the experiment when participants were still learning these contingencies. However, comparing the influence of reward on effort judgments between the first and second half of the task showed that there was no difference between these two halves for self- ($p>0.31$) and other-judgments ($p>0.28$). Importantly the effect of reward was already significant in the first half of the experiment (self-judgments: $p<0.001$; other-judgments: $p<0.001$). Taken together this indicates that participants had learned the contingencies between difficulty and rewards after training, before they started the experiment.

Results without applying exclusion criteria

In the main text we report results in which 11 subjects were excluded due to random effort ratings (their effort ratings were not significantly influenced by task difficulty, based on a linear regression with effort ratings as dependent variable and task difficulty as predictor), as well as excluded effort ratings that were ± 3 standard deviations from the subject's mean effort ratings.

These exclusion criteria were applied to ensure that only effort ratings that were not randomly chosen contributed to the analysis. However, here we show that the results are qualitatively similar when not excluding any subjects (using the full sample of N=62) nor outlier effort ratings from the analysis.

Across subjects, there was a significant influence of reward variation on effort estimation for self-judgments (one-sample signed test, $p<0.001$) as well as for other-judgments (one-sample signed test, $p<0.001$) and the effect of rewards on effort estimations was significantly stronger for other- than self-judgments (mean slopes of 0.12 and 0.09 respectively, Wilcoxon signed rank test, $p=0.028$). The amount of reward integration for self- and other-judgments was highly correlated across subjects ($r=0.75$, $p<.001$).

The comparison of the computation models showed that the Bayesian average model had the lowest BIC for self- (BIC=18079.2) and other-judgments (BIC=19119.8), followed by the flexible weighting model for self-judgments (BIC=18118.3) and the simple average model for other-judgments (BIC=19212.9).

Regarding associations of the integration of reward into effort judgments and conservative world-views, there was a positive association with the sum of the self- and other-judgment effect ($\beta=0.33$, $p=0.012$). This association between conservatism and reward influence could also be shown when separately considering self-judgments ($\beta=0.26$, $p=0.05$) and other-judgments ($\beta=0.35$, $p=0.008$).

With respect to whether or not the increased reward integration for individuals with more conservative attitudes was in line with Bayesian cue integration, we found a significant association between the weights of reward information (ω_r) predicted by the Bayesian averaging model and conservative world-views ($\beta=0.36$, $p=0.003$), indicating that more conservative participants integrated rewards more strongly due to higher uncertainty regarding the effort a person has exerted. When simultaneously entering the model-free and the model-based measures of reward integration as predictors of conservative attitudes, we found separate significant influences of ω_r ($\beta=0.41$, $p<0.001$) and the model-free measures of reward integration ($\beta=0.38$, $p=0.002$). This indicates that the strength of the influence of reward information on effort

judgments in conservative participants is over and above the predictions of the Bayes average model.

Influence of reward on effort ratings, controlling for within-difficulty fluctuations in gravity (due to trial-specific timing of presentation software)

Our main analysis for investigating the influence of reward on effort ratings focussed on a regression between variations of reward magnitude and effort ratings within difficulty levels (**Figure 2**). The rationale for this analysis was based on the fact that for each difficulty level rewards were drawn randomly and gravity (the velocity of the ball rolling backwards) was fixed. Therefore, reward magnitude should have been the only variable that could change across trials of the same difficulty level. However, due to differences in exact timing of the presentation software, even within each difficulty level there were slight fluctuations of the actual experienced gravity force, with the actual gravity level of some trials being marginally lower than intended. Although such within-difficulty fluctuations should theoretically only have added noise to the data (as they were unrelated to the received reward magnitude) and therefore decreased the observed effect of rewards on effort ratings, we wished to ensure that our results were not influenced by these variations. Therefore, we conducted the same regression between reward magnitude and effort ratings as shown in **Figure 2**, while controlling for the within-difficulty variation of trial-by-trial gravity force as a covariate (which had a significant effect on effort ratings for self-judgments: $p=0.01$, and other-judgments: $p=0.005$). As expected, when controlling for the within-difficulty fluctuations of gravity all results stay qualitatively the same, if anything with more pronounced effects. There was a positive influence of reward on effort ratings both for self- (one-sample signed test, $p<0.001$) and other-judgments (one-sample signed test, $p<0.001$) and this effect was stronger for other-judgments than self-judgments (mean slopes of 0.15 and 0.11 respectively, Wilcoxon signed rank test, $p=0.023$). These results indicate that our findings were not driven by within-difficulty variations of gravity force.

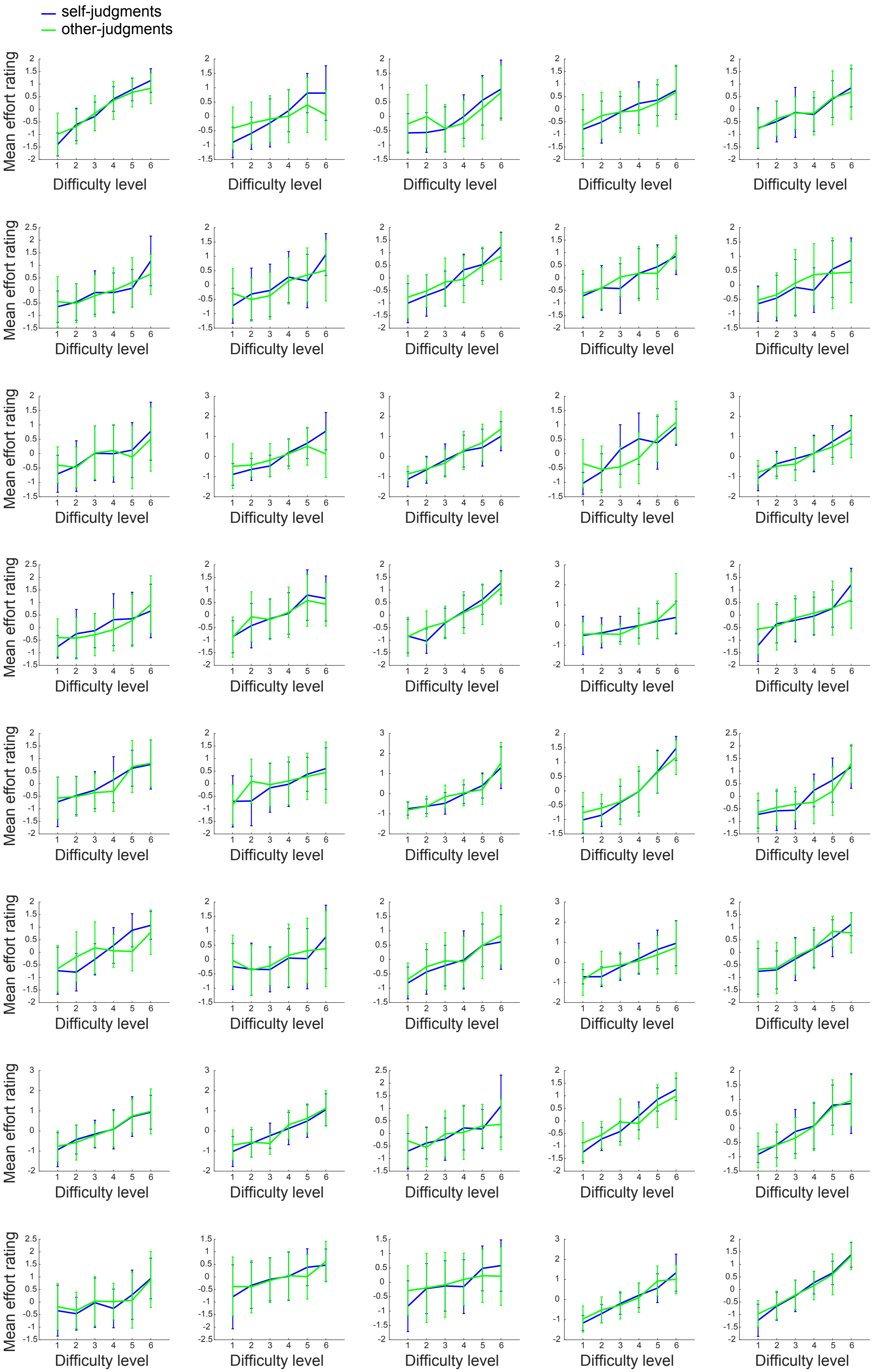

Figure continued on next page

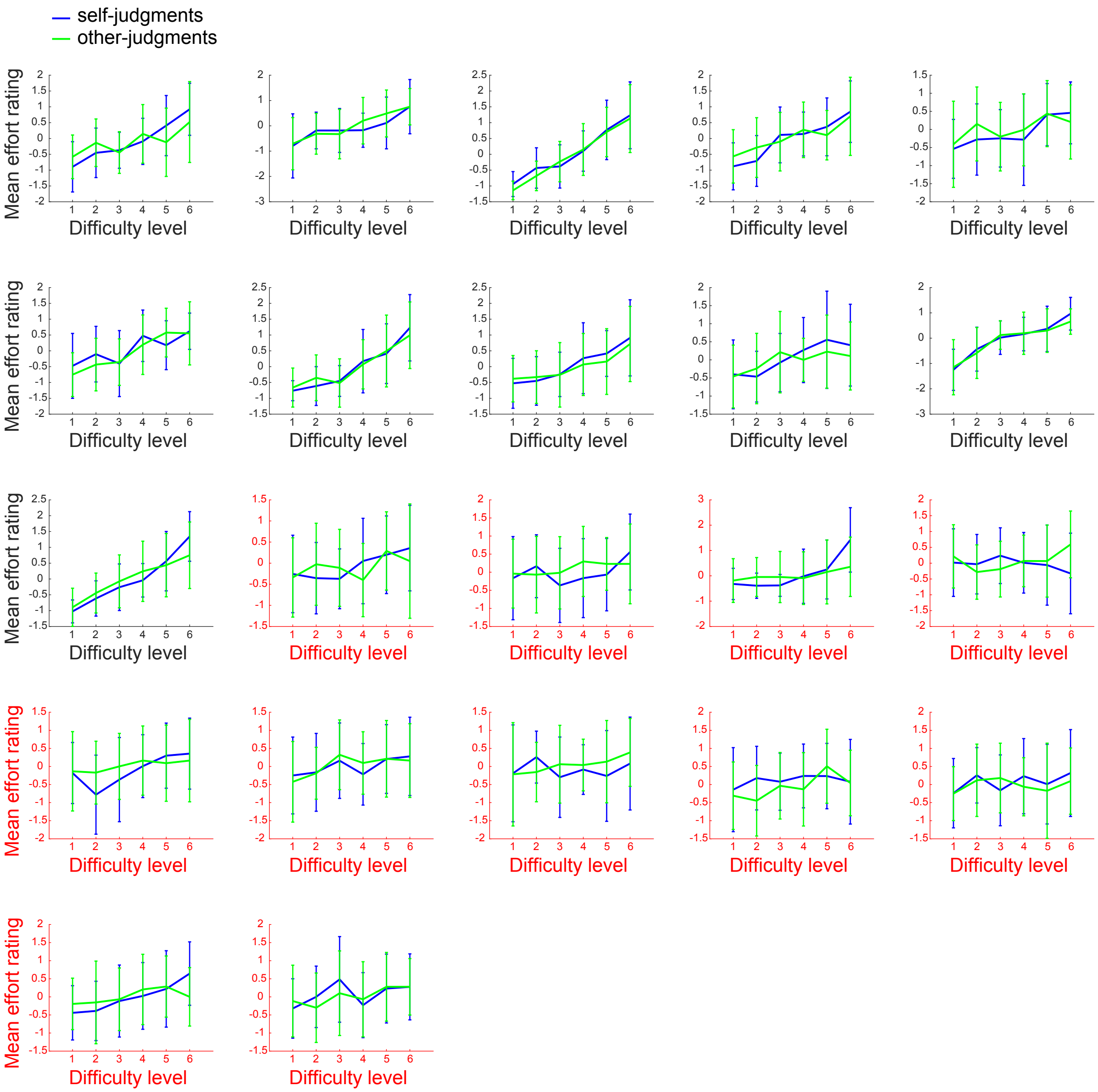

Supplementary Figure S1. **The psychometric plots showing the relationship between task difficulty and effort ratings for each subject (N=62), in trials where reward feedback preceded the effort rating.** Mean effort ratings (± standard deviation) for each difficulty level are shown, separately for self- (blue curves) and other-judgments (green curves). Eleven subjects that did not show a significant relationship between task difficulty and effort ratings were excluded from the final sample. Those excluded subjects are ordered at the bottom of the figure and marked by red coloured axes.

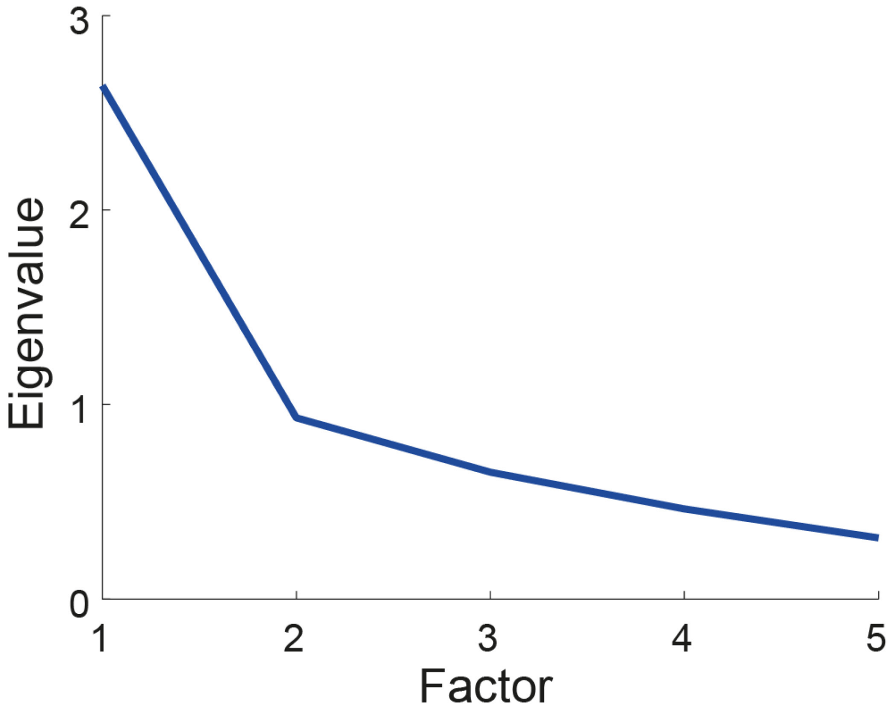

Supplementary Figure S2. **The scree plot shows that one factor is enough to capture the interrelation between several questionnaires used to assess conservative world-views.** Eigenvalues for up to five possible components are presented. There is a sharp decline in eigenvalue from the first to the second factor, forming a clear "elbow".

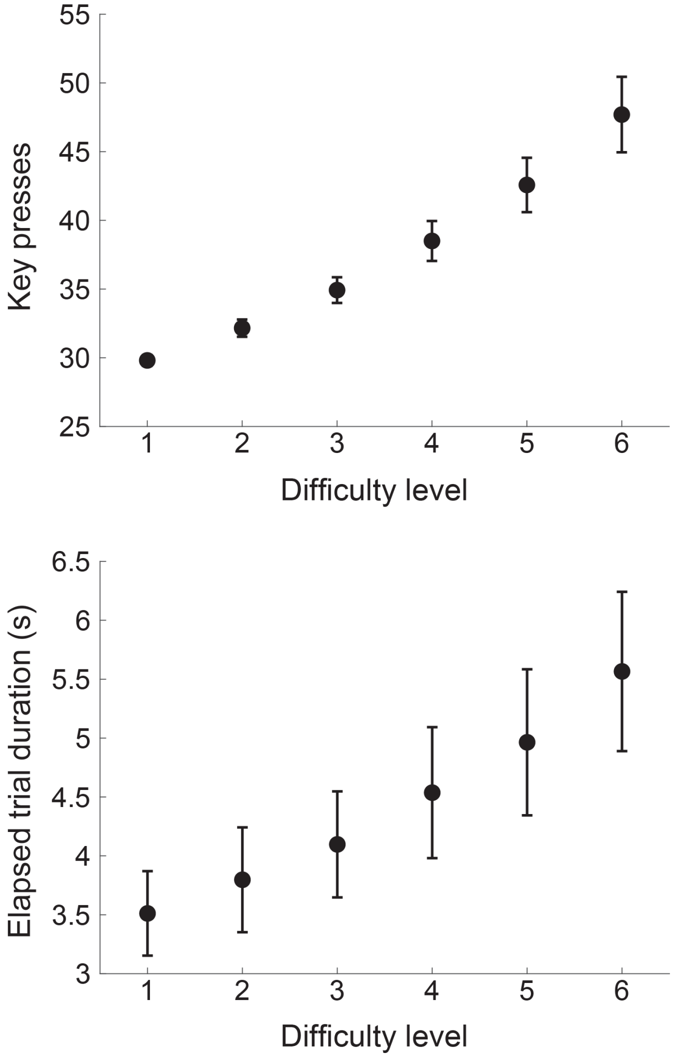

Supplementary Figure S3. **Psychometric plots showing the relationship between task difficulty, key presses and elapsed time. (A)** Group average of key presses for each difficulty level are shown (± mean of the within participant and within difficulty level standard deviation of key presses). **(B)** Group average of elapsed trial duration for each difficulty level are shown (± mean of the within participant and within difficulty level standard deviation of elapsed trial duration).

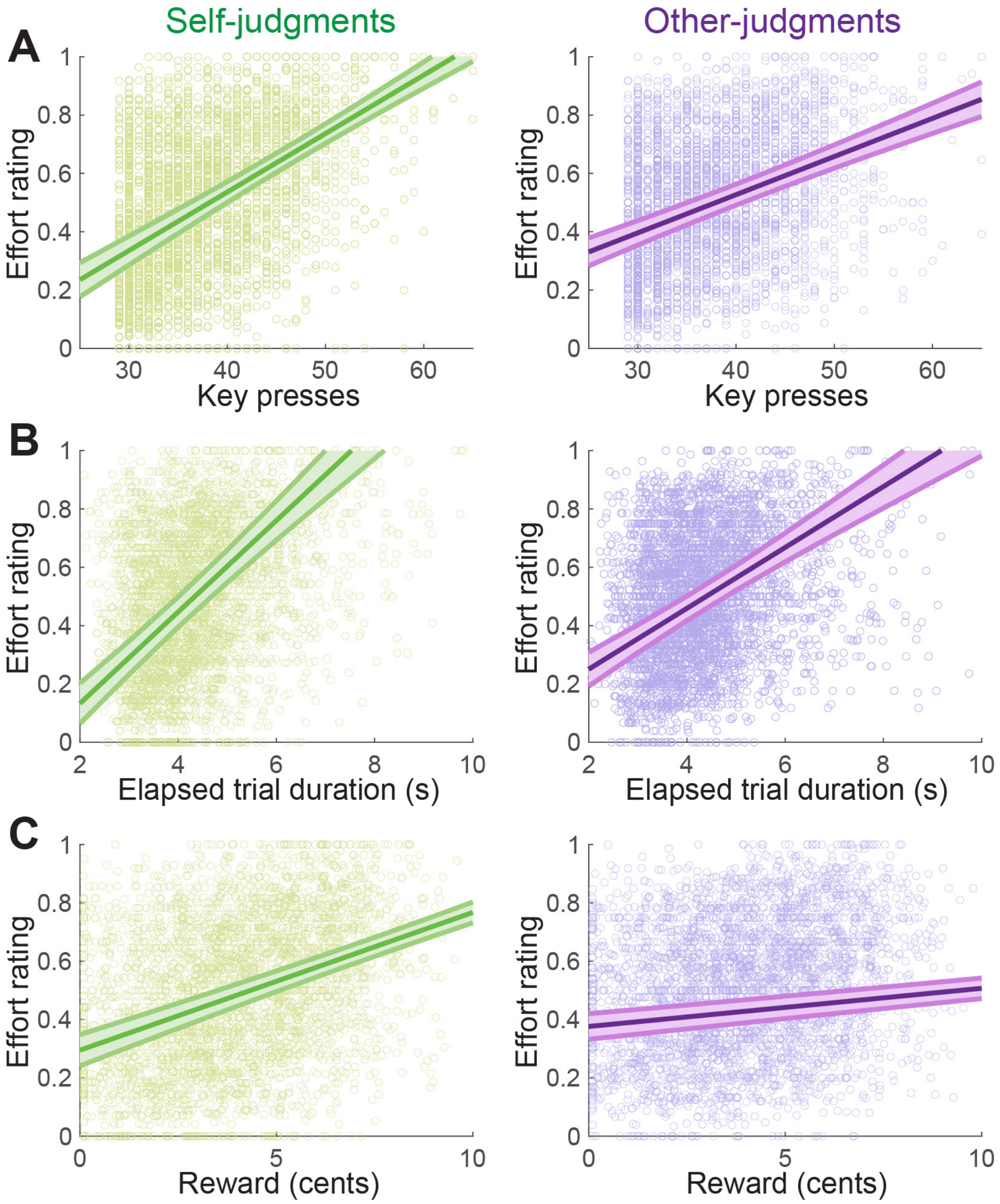

Supplementary Figure S4. **Multiple information sources are integrated to form a final effort rating. (A)** Relation between the number of key presses and effort ratings for self- (left) and other-judgments (right), based on the trials in which effort was rated *before* the reward was revealed (30% of all trials). Scatter plots (light colours) show the corresponding data pulled over all 51 subjects. The regression line shows the average slope for all subjects ± 95% confidence intervals of the individual slopes. **(B)** Similar to (A) for the relation between trial duration and effort ratings. **(C)** Similar to (A) for the relation between reward magnitude and effort ratings. Note that this relation effectively captures the correlation between reward and effort-rating built-in by the task design: since trials that are more difficult were associated with higher rewards and these trials were subjectively rated with higher exerted efforts, reward magnitude was related to the subjective estimations of exerted effort in these trials. The smaller slope for other-judgments is expected because there was a less tight link between actual difficulty and effort ratings (*cf.* A and B).
